# 

**Published:** 1862-01

**Authors:** 


### INDEX TO CONTENTS

OF

### HALL'S JOURNAL OF HEALTH,

### VOL. IX., 1862.

Bread, ....................78, 82, 225
Beans, .......................... 79
Buckwheat Cakes, ............... 79
Beef, .......................... 80
Bad Habits of Y., ............... 83
Bodily Emanations, ............. 141
Birds, Spare the, ............... 250
Bravery, Moral, ................. 252
Cookery, ....................... 78
Cornaro, ....................... 119
Constipation, .................. 123
Chloroform, .................... 124
Count Cavour, .................. 131
Chamber, Temperate, ............ 177
Church and World, .............. 254
Country and Town, .............. 202
Debt, ..................175, 185, 228
Dietetics, ..................... 227
Drunkenness, .................61, 249
Dispensary, A New, ............. 74
Eating and Drinking, ........... 116
Eight B's, ..................... 122
France in America, ............. 258
Glue, .......................... 82
Grapes, ........................ 113
Geology, ....................... 273
Hominy, ........................ 81
Health Education, .............. 123
High Living, ................... 131
Houses, Unhealthy, ............. 141
Health, Ruined, ................ 161
Hearing Preaching, ............. 269
Hidden Treasures, .............. 275
Longevity, ..................... 119
Mind Murders, .................. 67
Mush Biscuit, .................. 82
Music, Worcester's, ............ 167
Narrow House, .................. 70
Nervous Debility, .............. 178
Parsonages, .................... 99
Papered Chambers, .............. 144
Phonophorus, Stelles', ......... 269
Studio, ........................ 73
School-Children, ............... 105
Sleeping with Others, .......... 139
Sick Nation, ...........201, 223, 270
Soldier Health, ................ 233
Sickness Rate, ................. 252
Same Always, ................... 259
When Began We, ................. 75
William Wirt, .................. 249

The above are the Contents of Volume IX., for 1862, in addition to two hundred and twenty Health Tracts.

